# Bacterial infections and pathogenic situations: The case of *Staphylococcus lugdunensis*

**DOI:** 10.1080/21505594.2025.2543538

**Published:** 2025-08-13

**Authors:** Nicholas Binney

**Affiliations:** Institute of Ethics and History of Medicine, University of Tübingen, Tübingen, Germany

**Keywords:** Pathogen, commensal, pathogenicity, *Staphylococcus lugdunensis*, *Staphylococcus aureus*, pathogenic situation

## Abstract

Traditionally, researchers have viewed different species and strains of bacteria as pathogens or as commensals. Different bacterial species and strains may be placed on a spectrum of pathogenicity or virulence, with highly pathogenic bacteria at one end, and minimally pathogenic bacteria at the other. It has also been recognized that a species or strain of bacteria can behave sometimes as a pathogen, and at other times as a commensal. Consequently, researchers have proposed that a spectrum of less pathogenic to more pathogenic bacteriological *behavior* be produced, in contrast to the spectrum of less pathogenic to more pathogenic bacteriological *species*. I propose that a third spectrum of pathogenicity should be developed: a spectrum of *pathogenic situations*. This is illustrated using the example of *Staphylococcus lugdunensis*. Some researchers consider *S. lugdunensis* to be a commensal that might be used to eradicate the dangerous pathogen *S. aureus* from a patient’s nose, whereas other researchers consider *S. lugdunensis* to be a dangerous pathogen in its own right. Inoculating *S. lugdunensis* into the nose of a patient might be understood as inoculating them with a dangerous pathogen. However, thinking in terms of pathogenic situations, this same intervention could be understood as reducing the pathogenicity of the situation. As knowledge of the situations in which *S. lugdunensis* is dangerous is incomplete, this review of concepts of pathogenicity should not be used to justify challenge experiments, but rather to illustrate a different way of thinking about pathogenicity.

## Introduction

That it is difficult to divide bacteria into pathogens and commensal organisms is well recognized [1]. One attempt to address this problem is to place different species and strains of bacteria on a spectrum of pathogenicity that recognizes non-pathogenic organisms, as well as those of low, intermediate, and high pathogenicity. A common research project in infection biology is to attempt to match the degree of pathogenicity displayed by different species and strains of bacteria with the presence of various virulence factors expressed by those bacteria [2]. However, many researchers argue that bacterial pathogenicity is not simply a function of which virulence factors they harbour, but a function of many additional factors as well [3,4]. These include the host’s immune response, the site of infection, and the microbiological environment in which the bacteria exist. Consequently, some researchers have proposed a different spectrum of pathogenicity. Instead of a spectrum of more and less pathogenic *species* of bacteria, a spectrum of more and less pathogenic *behavior* can be produced [5]. Thus, the same species or strain of bacteria could appear on this spectrum several times, according to whether the infecting bacteria behave with no, low, intermediate, or high pathogenicity.

I propose a third spectrum of pathogenicity––a spectrum of *pathogenic situations*—to act as a counterpart of the spectrum of bacteriological behaviour in research. On this third spectrum, different situations are arranged from least to most pathogenic, where a pathogenic situation is one that promotes pathogenic behaviour in at least one of the bacterial strains found in that situation. Researchers can then match situations they believe to be pathogenic with the observed behaviour of the bacteria found in those situations. Furthermore, researchers can use this third spectrum of pathogenicity to describe clinical and research interventions from another perspective. Instead of making interventions to kill or control dangerous pathogens, researchers can instead intervene to make a situation less pathogenic.

I begin with a general discussion of these three different spectra of pathogenicity, describing what it means to be less or more pathogenic in each case. I close with a discussion of pathogenicity and *Staphylococcus lugdunensis*. Some researchers propose to investigate whether inoculating *S. lugdunensis* into the noses of patients can eliminate *S. aureus*. However, *S. lugdunensis* is viewed by many as a dangerous pathogen in its own right, meaning that such an intervention is seen as inoculating a dangerous pathogen into a person’s nose. Thinking in terms of pathogenic situations, this intervention might be understood differently––as either reducing the pathogenicity of the situation or leaving it unchanged. How spectra of pathogenicity are understood can have a profound effect on how medical interventions are understood, and therefore on whether they are considered reasonable to make. As knowledge of the situations in which *S. lugdunensis* is dangerous is incomplete, this review of concepts of pathogenicity should not be used to justify challenge experiments, but rather to illustrate a different way of thinking about pathogenicity. Although thinking about pathogenic situations should not supersede traditional ways of thinking about pathogenicity, it may be productive at times.

## Three spectra of pathogenicity

In bacteriology, the distinction between pathogens and commensals dates back to the late nineteenth century [1,6]. This distinction reflected the expectation that newly discovered species of bacteria would either be pathogens and always cause disease, or be commensal and always live harmlessly on, or even benefit, their host. This expectation did not remain intact for long [7–9]. Some strains of a bacterial species harmed the host, whereas other strains of that same species did not. Some hosts were harmed by a bacterial strain, and other hosts were not. Some environmental conditions promoted harmful infections, but others did not. These sources of variability confused the distinction between pathogens and commensals, as a species of pathogenic bacteria would not always harm hosts, and other species of commensal bacteria would not always live harmlessly on their host.

Despite these difficulties, the distinction between pathogenic and commensal bacteria has persisted. Bacteria are often deemed pathogens if they *at least sometimes* harm their host. Different types of pathogen have been described. Obligate pathogens must infect the host in order to complete their life cycle, whereas facultative pathogens can reproduce outside the host as well [10]. Primary pathogens can harm a perfectly healthy host, whereas opportunistic pathogens can only harm a host whose defences against infection have been compromised [1]. Accidental pathogens have properties that allow them to live in the environment outside the host, but if the microbes enter the host, these same properties can harm the host and result in infection [11,12].

In wealthy countries – in contrast to many parts of the world – the incidence of infections caused by obligate pathogens has reduced dramatically over the twentieth century [13]. However, the use of implanted devices such as intravenous catheters, prosthetic joints, and prosthetic heart valves in elderly, immune-compromised, or otherwise vulnerable patients has increased considerably in recent years [13]. This has produced circumstances in which bacteria that once were understood as commensals can infect their host and produce serious disease [4]. Furthermore, the type of immune response mounted by the host can influence the pathology produced during an infection [14,15]. Indeed, in some cases pathology is produced largely by the host’s immune system – or by some combination of immune response and microbe – rather than solely by the activity of the microbe [14,15]. If the distinction between commensals and facultative opportunistic or accidental pathogens depends on the properties of the host, then the distinction between commensals and pathogens as types of microbe becomes difficult to maintain.

Rather than maintaining a strict distinction between pathogenic and commensal species of bacteria, continuous concepts such as *virulence* and *pathogenicity* have been developed. These place bacteria on a continuum from pathogen to commensal, according to both the *severity* of harm caused and the *frequency* with which harm is caused. Formally, pathogenicity is not a continuous concept [1]. If a bacterium can cause harm, then it has pathogenicity; if it cannot, then it does not have pathogenicity. The concept of virulence was developed as a continuous complement to pathogenicity, with highly virulent bacteria being able to cause severe harm to their host, and bacteria with low virulence being able to cause only slight harm to their host [7]. The words pathogenicity and virulence “are often used interchangeably, but pathogenicity has been defined as the ability of an organism to cause disease and virulence as the relative severity of the disease caused by the organism” [16]. However, if it is recognized that even commensal bacteria can on occasion harm their host, then the distinction between bacteria with and without pathogenicity collapses. Furthermore, highly virulent bacteria that are capable of causing severe harm or even death are also often referred to as highly pathogenic microorganisms [17–20]. Bacteria are also described as having high, medium, and low pathogenicity, depending on the severity of the harm they can cause [21–23]. Consequently, I do not make the distinction between pathogenicity and virulence here, recognizing that pathogenicity has several different meanings, including one synonymous with virulence.

Pathogenicity is sometimes defined as the frequency with which a microorganism harms its host by causing symptomatic infection. “Pathogenicity is a measure of the proportion of infections resulting in overt disease” [24]. An intrinsic pathogenicity index (IPI) for microorganisms has been developed, which is “the ratio between the number of patients infected by a particular microorganism and the number of patients who carry the identical microorganism in the throat and/or gut” [25, 26]. This ratio is used to place microorganisms on a scale of pathogenicity, from high-level pathogens, with an IPI close to 1, to low-level pathogens, with an IPI close to 0 [25,26]. Alternatively, others have proposed to quantify the *pathogenic potential* of a microorganism using the ratio of the fraction of symptomatic patients to the size of the infecting inoculum [27,28]. This recognizes that patients exposed to larger numbers of microorganisms are more likely to become symptomatic. These are efforts to quantify the pathogenicity of a microorganism according to the frequency with which it causes harm, thus placing different species on a spectrum of pathogenicity.

Concepts of pathogenicity based on severity of disease and frequency of disease are sometimes combined in indices. In such cases a bacterium would have high pathogenicity if it *frequently* causes *severe* harm or death, such as in the intravenous pathogenicity index used in poultry farming [29].

**Figure 1.**
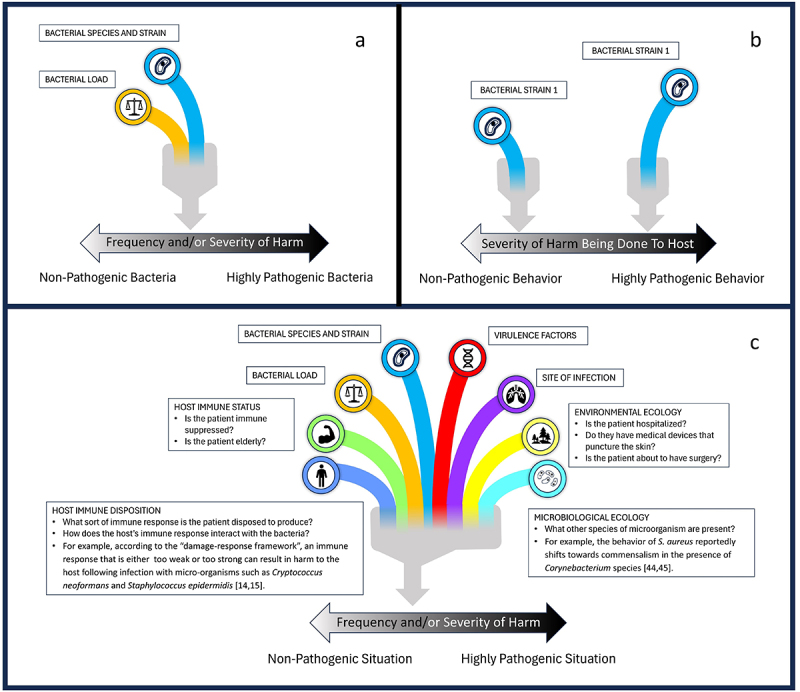
Three spectra of pathogenicity. The first spectrum (1a) arranges different species and strains of bacteria on a spectrum according to the frequency with which infection with the bacterium harms the host and/or the amount of harm that the bacterium is capable of doing, sometimes taking bacterial load into account. Each bacterium can only appear on this spectrum once. The second spectrum (1b) places bacteria on a spectrum according to the harm that is being done to the host in any particular infection. The same species and strain of bacterium can appear on the spectrum more than once, being non-pathogenic when they do not cause harm and pathogenic when they do. The third spectrum (1c) arranges different situations involving a bacterium on a spectrum according to the frequency with which the situation is expected to cause harm and/or the severity of harm expected to occur. Each situation is defined using a range of relevant factors, including bacterial species and strain, the bacterial load, the virulence factors present, the site of infection, host immune status and disposition, the microbiological ecology of the host and their wider environment.

This, then, is the first spectrum of pathogenicity (Figure 1(a)). Different species and strains of bacteria are placed on a spectrum according to the severity of harm they may cause, and according to the frequency with which they cause harm, or some combination of the two.

It is worth reflecting for a moment how to think about the situation of a person colonized by two potentially pathogenic bacteria. How should the probability and severity of the illness be estimated? Should the pathogenicity of the two bacteria be treated as two independent events, such that the two probabilities of illness can be added together to estimate the probability of suffering from illness caused by one or the other bacteria? This question will be considered again in the discussion of Staphylococcal infections below.

In addition to observing the probability and severity of illness following infection with different species and strains of bacteria, researchers also try to explain why some species and strains of bacteria frequently cause severe harm but others do not. Researchers try to match the presence and absence of *virulence factors* and *virulence genes* to species and strains of bacteria implicated in disease and to the presence and absence of illness. They also try to explain mechanistically how virulence factors enable bacteria possessing such factors to cause disease. For example, researchers have investigated the role that clusters of genes called *pathogenicity islands* play in the variable pathogenicity of different strains of *Escherichia coli* bacteria, which range from gut commensals to highly pathogenic intestinal and extraintestinal strains [30,31]. Pathogenicity islands are more common in strains of *E. coli* that regularly cause serious disease than they are in strains of bacteria that tend to be commensal [32,33]. Pathogenicity islands are also associated with patient mortality in clinical data [31]. Targeted mutagenesis of these genes can reduce mortality in animal models of infection [32,34]. However, pathogenicity islands and their associated virulence factors are often found in strains of commensal bacteria, albeit often at lower frequencies than in pathogenic bacteria [32,35–37]. It is difficult to distinguish those factors that allow bacteria to adopt a commensal lifestyle from those that allow them to colonize sites where they might act as pathogens. It is also difficult to distinguish both of these from the factors that are directly implicated in harming the host. It is challenging to distinguish *pathogen-specific* virulence factors from *common* virulence factors. It is difficult to distinguish *true* virulence factors from *niche* virulence factors [36,38,39]. Some researchers doubt that virulence can be understood as an attribute of bacteria, as the behaviour of bacteria will depend at least on the attributes of the host as well [3,4]. Whether a gene or some other factor produces pathogenicity may well depend on many things in addition to the attributes of the bacterium. “Currently, bacterial traits are subject to a binary categorisation whereby some are labelled as virulence factors while others are not. We demonstrate that traits’ effects on virulence are anything but binary. Rather, they strongly depend on the infection context” [2, see also 40,41]. Explaining the variable pathogenicity of different strains and species of bacteria using virulence factors is challenging [42].

An alternative approach understands the distinction between pathogens and commensals differently. Instead of the species or strain of bacteria dictating their identity as either pathogens or commensals, some researchers argue that bacteria belonging to a particular species or strain might sometime *behave as* pathogens, and at other times as commensals [5]. Here, it is the *behavior* of the bacteria in a particular infection that dictates whether they are pathogens, not their species or strain. That behaviour is not the simple result of bacterial genetics but rather is an emergent property of many interacting factors [43–45]. Virulence can be understood as the *outcome* of these interactions rather than as the input to them provided by the bacterium [1,46]. Researchers point to a continuum of commensal versus pathogenic bacterial behaviour, which ranges from *mutual adaptation between microbe and host* (and *tolerance*), to *mutual offence between microbe and host* (and *inflammation*) [5]. A variety of factors, including the state of the microbiome in which the bacterium lives, the immune status of the host, the species and strain of the microbe, and its virulence factors, act in concert to determine whether a bacterium behaves as a pathogen or as a commensal [43,47]. Here, to be a pathogen is *to behave* as a pathogen harming its host rather than to be a certain species or strain of bacteria. Pathogenic behaviour can be quantified in terms of the amount of damage done to the host by a particular infection, rather than by the amount of damage that species of bacteria is capable of doing.

Here, then, is the second spectrum of pathogenicity, which places bacteria (perhaps of the *same* species and strain) on a continuum from behaving-like-a-commensal to behaving-like-a-pathogen (Figure 1(b)).

A third spectrum of pathogenicity should be described. Even if we recognize the variation in behaviour of bacteria of the same strain or species, we need some way to describe the situations that cause pathogenic behaviour, so that this behaviour can be explained. As discussed, to explain the degree of pathogenicity or virulence of different species and strains of bacteria, researchers attempt to match different species and strains of bacteria with virulence factors. Similar matchmaking practices are visible in cancer research. Tumours can often be placed on a continuum from entirely benign, through slow-growing and only locally invasive, to fast-growing, spreading to distant sites and causing profound cachexia. Even so, physicians still need pathological criteria for tumour classification, to predict and explain the clinical behaviour of the tumour. What is the equivalent matchmaking project in research that considers pathogenicity as a spectrum of bacteriological behaviour? I propose that these criteria should describe the *pathogenic situation* of the host. The description of the pathogenic situation should include the species and strain of bacterium present on the host, the bacterial load, any relevant virulence factors, its location in the host, its microbiological environment, the immune status of the host, and any other factor deemed relevant. Pathogenic situations might range from non-pathogenic, in which no harm is expected to come to the host, to minimally, moderately, and highly pathogenic, where considerable harm to the host is expected without intervention (Figure 1(c)). The species or strain of bacterium present on the host might be enough to determine the pathogenic situation [4]. Some species of bacteria never harm the host, whereas others regularly harm hosts with well-functioning immune systems. Much of the time, however, other factors, such as the host’s immune status and the microbiological environment, play an important role in determining bacterial behaviour [4,44,47], and need to be described to determine the pathogenic situation.

In many cases, however, researchers and clinicians have limited ability to determine which situations are pathogenic. Not enough is known about the interactions between pathogens/commensals and the host/host immune system to explain the pathogenic behaviour of many bacteria [5]. Thus, it is important to recognize that the pathogenic situation of many patients will be uncertain. Researchers should not feel compelled to say that a patient is in a non-pathogenic, minimally pathogenic, moderately pathogenic, or highly pathogenic situation; they should recognize that a patient may also be in an uncertain pathogenic situation. Uncertain pathogenic situations can serve as opportunities for investigation, so that researchers and clinicians can develop a more complete knowledge of what causes a situation to be pathogenic.

I have described three different ways of understanding the distinction between pathogens and commensals. The first places *different species or strains of bacteria* on a continuum from commensal to pathogenic, with pathogens being those species or strains that can cause harm to the patient. The second places the *behavior of bacteria* (perhaps of the same species or strain) on a continuum from commensal to pathogenic, with pathogens being those bacteria that behave like a pathogen by harming their host. The third places the *situations in which patients find themselves* on a continuum from non-pathogenic to highly pathogenic, according to whether the bacterial species, their virulence factors, their location, their microbiological environment, and the host immune response are expected to result in harmful bacteriological behaviour. Research into infection biology may proceed by matching different combinations of virulence factors to the strains and species of bacteria found at different positions on the first spectrum. Research may also proceed by matching the behaviour of bacteria to different pathogenic situations, gradually building up knowledge of which situations are highly pathogenic, moderately pathogenic, and minimally pathogenic.

These may seem like subtle distinctions, but they make an important difference to medical and research practice. This can be illustrated by considering infection with Staphylococci, and especially whether inoculating a person’s nose with *S. lugdunensis* produces a more pathogenic situation or a less pathogenic situation.

## *Staphylococcus aureus*, *Staphylococcus lugdunensis*, and pathogenic situations

The traditional way of classifying Staphylococcal bacteria is into coagulase-positive Staphylococci (CoPS), such as *S. aureus*, and coagulase-negative Staphylococci (CoNS), such as *S. carnosus*, *S. epidermidis*, and *S. lugdunensis* [23,48,49]. This is not a phylogenetic classification. Coagulase was chosen to distinguish different species of Staphylococcus because it was thought to distinguish between the pathogenic and non-pathogenic species [48]. Until the 1980s, only CoPS were considered important opportunistic pathogens, whereas CoNS were regarded as harmless commensals [22,48–51].

*S. aureus* is one of the most important opportunistic bacterial pathogens of humans [52,53]. In 2019, *S. aureus* was associated with more than a million of the estimated 13.7 million infection-related deaths globally, making it the leading bacterial cause of death in 135 countries [54]. It is the most frequent source of skin infections globally [55], and one of the leading causes of pneumonia, surgical site infections, prosthetic joint infections, life-threatening bloodstream infections, and cardiovascular infections [56]. The morbidity and mortality associated with *S. aureus* has been described as “staggering” [57].

*S. aureus* can enter host tissues and even the bloodstream through surgical and accidental wounds, as well as via indwelling medical devices [53]. Topical antibacterial and antibiotic treatments are effective, but recolonization with *S. aureus* after three months is common, and concerns about antibiotic resistance are growing [58]. Alternative methods of decolonizing *S. aureus* that provide long-lasting effects, without leading to antibiotic resistance or disrupting the microbiota, are desired [58]. Decolonization may help prevent acute infections, or help treat people with chronic *S. aureus* infections of the skin, for example [58].

Rather than using *antibiotics* to kill *S. aureus*, a possible alternative approach is *probiotic*. The nares are the main carriage site for *S. aureus*, which acts as a reservoir for these bacteria and as a source of infection [59]. Bacteria that compete with and kill *S. aureus* might be inoculated into a patient’s nose and may eradicate *S. aureus* from this site. The beneficial role CoNS can play as commensal organisms in the health of their host is widely acknowledged, not least because they help limit *S. aureus* colonization [60,61]. *S. lugdunensis* is known to produce the substance “lugdunin,” which kills *S. aureus* cells in vitro [62,63]. Because it is frequently found living on the skin of healthy people, *S. lugdunensis* is often described as a commensal organism [63–66]. “*Staphylococcus lugdunensis* is a coagulase-negative staphylococci that is considered normal skin microbiota” [66]. Approximately 30% to 50% of the general population are asymptomatic carriers of *S. lugdunensis*, which is most frequently found in the groin, axilla, and nail bed [67–69]. *S. lugdunensis* is also found in the nares of 6% to 9% of the general population, and people who carry *S. lugdunensis* are six times less likely to carry *S. aureus* in their nares than people who do not [62,63,70,71]. This has prompted researchers to suggest that inoculating *S. lugdunensis* into the nose may be able to decolonize the nose of *S. aureus* and provide sustained protection from infection [62,65,71]. The idea is to use commensal bacteria to eliminate pathogenic bacteria from a niche and prevent infections.

## The strict division of Staphylococcal bacteria into pathogens and commensals is not sustainable

However, the simplistic notion that *S. aureus* is a pathogen and *S. lugdunensis* is a commensal is not sustainable. Around 30% of the healthy population are intermittent or persistent carriers of *S. aureus* [59]. Consequently, it is often described as a normal commensal of the human body, even though its role in causing severe disease is acknowledged [72–77]. “*Staphylococcus aureus* is a commensal organism that is widely distributed in nature. Approximately 20%–30% of healthy people carry this organism, mostly in the nose” [72]. So, although *S. aureus* is described as a serious pathogen, it is also described as a commensal organism.

The same can be said of *S. lugdunensis*. Although CoNS are traditionally seen as harmless, and continue to be described as commensals [61,74], they are increasingly recognized as important pathogens, especially in hospitalized patients with medical devices that penetrate the skin [22,23,50,51,78,79]. Recent papers that describe *S. lugdunensis* as a commensal also describe it as a pathogen [64–66]. Like *S. aureus*, *S. lugdunensis* is known to cause skin and soft tissue infections, subcutaneous tissue infections, bone and joint infections, prosthetic joint infections, vascular catheter-related infections, infective endocarditis, bacteraemia, and abscesses [80]. “*Staphylococcus lugdunensis* is an aggressive pathogen that shares major virulence factors with *S. aureus*” [81]. In particular, *S. lugdunensis* is known to cause a very aggressive form of infective endocarditis. As *S. lugdunensis* is a CoNS that can cause very serious disease, it has been described as a “wolf in sheep’s clothing” [65,80,82]. “*Staphylococcus lugdunensis* is an emerging high-virulent pathogen” [80].

As is the case for *S. aureus*, the ability of *S. lugdunensis* to *behave* both as a commensal and as a pathogen is recognized [83,84]. These conflicting descriptions of CoPS and CoNS as commensals and as pathogens again reveal the problem with considering different species of bacteria either as pathogens or as commensals.

## Do *S.* *aureus* and *S.* *lugdunensis* exist on a spectrum of pathogenicity?

One response to this problem is to present different species of *Staphylococcus* on a spectrum of pathogenicity or virulence. Species of CoNS such as *S. carnosus* are considered as non-pathogenic, *S. epidermidis* as of medium pathogenicity, *S. lugdunensis* as of medium or high pathogenicity, and CoPS such as *S. aureus* as highly pathogenic [21,23,48,85,86].

Consideration of the degree to which bacteria are pathogenic extends beyond the level of species to different strains of those species as well [48,87]. Different strains of *S. aureus* may be considered as more or less pathogenic, according to the harm they do, the frequency with which they cause harm, and the virulence factors they harbour [88–90]. Virulence factors may allow adhesion to host cells, produce toxins, assist in evading the immune system, or develop antibiotic resistance. *Staphylococcus aureus* has an open pangenome, and strains regularly add new genetic material and virulence factors to their repertoire [91]. However, strains implicated in serious disease (such as USA300 in the US) are among the most common found living innocuously in the general population [71,89,90,92]. *S. lugdunensis* shares many virulence factors with *S. aureus*, distinguishing it from other CoNS [65,93]. In contrast to *S. aureus*, *S. lugdunensis* has a closed pangenome, and has systems to prevent the regular uptake of new genetic material [93,94]. “Its virulence in clinical settings does not rely on its ability to acquire and exchange antibiotic resistance genes or other virulence factors as shown for other staphylococci” [94]. With this in mind, it is possible to consider how pathogenic these bacteria are in terms of the severity of harm they cause and the frequency with which they cause it.

## How pathogenic are *S.* *aureus* and *S.* *lugdunensis*?

Both *S. aureus* and *S. lugdunensis* can cause lethal bacteraemia and endocarditis [95–98]. *Staphylococcus lugdunensis* can produce a very aggressive infective endocarditis, with a mortality rate estimated at 40% or higher [96,99,100]. Mortality from *S. aureus* infective endocariditis is estimated at between 20% and 40% [95,98,101,102], making *S. lugdunensis* endocarditis even more lethal than *S. aureus* endocarditis. Like *S. aureus*, but unlike other CoNS, *S. lugdunensis* has a propensity to infect native heart valves in addition to prosthetic valves and other implanted devices [96,99,100]. Both *S. aureus* and *S. lugdunensis* are highly pathogenic or virulent in the sense of being able to cause severe harm.

However, *S. lugduneneis* causes serious infections less frequently than *S. aureus*. The incidence of *S. lugdunensis* infections has been underreported [64,84,103]. Some *S. lugdunensis* produce a bound coagulase that can lead to misidentification as *S. aureus*, and some laboratories have not routinely distinguished between different species of CoNS. The increasing notoriety of *S. lugdunensis*, and the widespread use of diagnostic technology, such as polymerase chain reaction and matrix-assisted laser desorption ionization time-of-flight mass spectrometry, help to address this issue [103,104]. The incidence of clinically significant *S. lugdunensis* bacteraemia among hospital admissions is low [97,103–105]. One Korean study found only 15 cases in over a million admissions [97]. An American study found 75 cases of *S. lugdunensis* bacteraemia in over 3,000 hospitalized cases [103]. In contrast to this, the incidence of *S. aureus* bacteraemia is high, at between 20 and 50 cases per 100,000 people in the *general population* [106,107]. There were 120,000 cases of *S. aureus* bacteraemia in the US in 2017, and 20,000 deaths [108]. The mortality rate from *S. aureus* bacteraemia (10% to 30%) [56,95] and *S. lugdunensis* bacteraemia (10% to 45%) [95,97,109] may be similar, but *S. aureus* bacteraemia is responsible for more deaths because it is more frequent. The same is true for infective endocarditis. A ten-year study in Sweden found that in 2184 cases of infective endocarditis, 1892 were caused by *S. aureus*, and 30 by *S. lugdunensis* [99].

So, in one sense, *S. lugdunensis* is *less* pathogenic than *S. aureus*, because it causes infections much less frequently than *S. aureus*. In another sense, *S. lugdunensis* is *more* pathogenic or virulent than *S. aureus*, because it can cause infections that are more aggressive and have a higher mortality rate than *S. aureus*. Given this, why would exposing the patient to another virulent pathogen be entertained as a potential therapy for *S. aureus* colonization? “*S. lugdunensis* is being investigated as a probiotic to eradicate *S. aureus* from the nares of carriers. However, this is contraindicated by its innate virulence” [65]. Much depends on whether *S. lugdunensis* is seen as a pathogen, a commensal, or something in between––or as a part of a pathogenic situation. Researchers could think in terms of the pathogenicity of the situation the bacteria produce, rather than the pathogenicity of the bacteria they introduce. Researchers could try to identify pathogenic situations. To do these, however, more information is needed about the situations in which these bacteria may prove harmful.

## Do we know which situations are pathogenic?

The main source of *S. aureus* found in infections is the patient’s own nose [53,110–114]. “Indeed, the majority of patients with severe, invasive disease are infected by the *S. aureus* residing in their noses” [76]. The source of serious infection for *S. lugdunensis*, by contrast, is often unknown [96,97,115,116]. Intravenous catheters and other implanted devices can provide a route of entry to the bloodstream for Staphylococci in hospitalized patients [48,117], but there are perfectly healthy, young people who develop *S. lugdunensis* endocarditis in the community with no indication of the route by which they were infected [97,118]. Like *S. aureus*, *S. lugdunensis* endocarditis is often a community-acquired infection [96,99,119,120]. But, when a portal of entry has been identified, it is mainly associated with procedures involving a puncture or incision to the skin in the groin. “When *S. lugdunensis* has been implicated by percutaneous portal of entry, it has been related mainly to groin procedures, as perineal skin is an area where *S. lugdunensis* will preferentially reside” [100]. Case reports that provide a suspected source of infection commonly describe patients having recently undergone a vasectomy, or who have had a femoral artery catheter placed, especially for angiography or angioplasty, or who have a lesion to the groin [96,115,116,121]. Shaving the groin before a surgical procedure is identified as a risk factor for infection with *S. lugdunensis* [84]. “All these clinical reports strongly suggest that pubic-inguinal area carriage is the source for iatrogenic *S. lugdunensis* infections” [122]. People who have had knee operations and prostheses are also commonly represented [96,115,116]. Indeed, a significant majority of *S. lugdunensis* infections occur below the waist [84,123]. Those infections that occur above the waist are often breast abscesses [84,124,125]. These observations are consistent with the groin, armpits, and toes being preferred sites for *S. lugdunensis* [67]. Although knowledge of the circumstances that cause *S. lugdunensis* to become pathogenic is incomplete, available information indicates that serious infections tend to arise from wounds, punctures, or lesions to the groin, armpit, or lower body that might pass bacteria from those sites into the bloodstream. In contrast to *S. aureus*, nasal carriage is not identified as an important source of infection for *S. lugdunensis*.

Given these considerations, we can speculate about situations that might qualify as pathogenic. Consider a person enrolled in a trial to determine if inoculating *S. lugdunensis* into the nose can decolonize the nose of *S. aureus*. Because *S. lugdunensis* is capable of causing serious disease, experimental protocols for inoculating this species into participants’ noses should be designed with care.^1^ One particular concern is that *S. lugdunensis* might be inhaled and have the opportunity to cause infection in the lung. This possibility can be explored using animal models. The nasal passage of mice might be inoculated with a solution containing 10^7^ to 10^9^ colony forming units (CFU) of *S. lugdunensis* in a volume greater than the nasal passage can hold, leading to aspiration into the lung. If no harm comes to the mice, it would suggest that the risk of harm from aspiration of *S. lugdunensis* into the lung in humans might also be low. Human challenge experiments could begin by inoculating 10^4^ CFU of human-derived, lugdunin-producing, antibiotic-sensitive *S. lugdunensis* into the nose of healthy volunteers, gradually increasing this to 10^8^ CFU. It is worth noting that experiments have already been carried out in which 10^7^ to 10^9^ CFU of *S. aureus* was inoculated into the nose of healthy volunteers, without harmful effects [126–128].

With further research, it may be possible to use targeted mutagenesis to produce less virulent strains of *S. lugdunensis*, which may be candidates for probiotic use. Other elements that comprise a pathogenic situation can also be considered. If the nose is not an important source of severe *S. lugdunensis* infections, then *S. lugdunensis* colonizing the nose may do little to make the situation of a patient more pathogenic, even though the patient’s nose is now colonized by bacteria that can act as a dangerous pathogen.

A person may, by touching their nose and then other parts of their body, transfer the bacteria to their groin, which might make their situation more pathogenic. However, because *S. lugdunensis* is a rare cause of serious infections, and is found living on up to half of the general population, it is questionable whether the situation will become much more pathogenic following its colonization of the skin. This may be especially true for people who already have *S. lugdunensis* living on their skin before the bacteria were inoculated into their nose.

Furthermore, if the person is not anticipated to undergo a vasectomy, femoral artery catheterization, or knee surgery, and if they do not have lesions to their perineal region, this limits the opportunity for the bacteria to enter the bloodstream, and this diminishes the pathogenicity of the situation. People enrolled in this hypothetical trial may be healthy and not scheduled to undergo any such procedure.

Finally, those enrolled in this trial *already have S. aureus living in the nose*. If it causes bacteraemia or infective endocarditis, *S. lugdunensis* can be even more dangerous than *S. aureus*, but *S. aureus* causes serious harm much more frequently than *S. lugdunensis* does. If *S. aureus* is inoculated into the nose of a person already colonized by *S. aureus*, then the situation may not become more pathogenic. A new pathogen has not been added to the nose, and the bacterial load in the nose may not be increased significantly following colonization [126]. If *S. lugdunensis* caused disease of the same severity with the same frequency, and if *S. lugdunensis* occupied the same pathological niche as *S. aureus*, causing similar disease in a similar way without interacting with *S. aureus*, then this would be equivalent to inoculating *S. aureus*, and the pathogenicity of the situation would be unaltered. But *S. lugdunensis* causes disease less frequently than does *S. aureus*, so colonizing the nose with these bacteria should not increase the pathogenicity of the situation. Indeed, the hope is that the *S. lugdunensis* should *replace* the *S. aureus* in the person’s nose, so that the bacteria that cause disease less frequently remain in the nose. Arguably, the situation would become less pathogenic than it was, should *S. lugdunensis* replace *S. aureus*.

If we think about pathogenicity as a property of a bacterial species, then inoculating *S. lugdunensis* into the nose of a person may be to inoculate them with a moderately to highly dangerous pathogen. However, if we think about pathogenicity as a property of a pathogenic situation, then inoculating *S. lugdunensis* into the nose of a person may reduce the pathogenicity of the situation or leave it unchanged. Much depends on how we understand pathogenicity. Importantly, as the circumstances under which *S. lugdunensis* produces life-threatening disease are often unknown, researchers may decide that the pathogenicity of situations involving *S. lugdunensis* are uncertain.

## Conclusion

It is common to think about different species or strains of bacteria as having no, low, medium, or high levels of pathogenicity or virulence. This might mean that a certain strain of bacteria frequently causes harm to its host, or that it causes severe damage when it does harm its host, or some combination of the two. Researchers frequently try to explain differences in pathogenicity using virulence factors found in species or strains of bacteria.

The pathological behaviour of bacteria can depend on factors other than species, strain, and virulence factors. Recognizing this, some researchers have proposed that the spectrum of pathogenicity be understood differently: as a spectrum of pathogenic *behavior*, rather than a spectrum of pathogenic *species*. I have argued that a third spectrum of pathogenicity – a spectrum of *pathogenic situations*—is also useful to recognize. Researchers can investigate which situations promote the pathogenic behaviour of bacteria, to give clinicians the ability to identify and address pathogenic situations.

I have used the example of *Staphylococcus lugdunensis* to illustrate how this third spectrum could change how pathogenicity is understood. Researchers have proposed inoculating *S. lugdunensis* into the nose of people carrying *S. aureus*, in the hope that the *S. lugdunensis* will outcompete and eradicate the *S. aureus* from the nose. However, *S. lugdunensis* is understood by many clinicians and researchers to be a dangerous pathogen, capable of causing fatal diseases such as endocarditis, and it may be deemed unethical to inoculate dangerous pathogens into the noses of patients. Much is unknown about the situations in which *S. lugdunensis* is dangerous, and this review should not be used to justify challenge experiments. Even so, what is known about *S. lugdunensis* can be used to illustrate how this intervention might be understood differently. Although *S. lugdunensis* can cause severe disease, it does so much less frequently than *S. aureus* does. Because these patients already have *S. aureus* in their nose, even if *S. lugdunensis* does not eradicate *S. aureus*, the risk of serious disease may well not rise following its inoculation. Furthermore, serious cases of *S. lugdunensis* infection tend to arise from wounds or other lesions to the lower body, and not from nasal colonization. Colonizing the nose, especially in people who already have *S. lugdunensis* living in their lower body, may do little to increase the frequency of serious *S. lugdunensis* infection in these patients. If the *S. lugdunensis* does eradicate the *S. aureus*, then a pathogen that causes severe harm more frequently can be eradicated from the site from which it often causes serious infections. In other words, the introduction of a potentially dangerous pathogen may make the situation less pathogenic. When it is uncertain which situations are pathogenic, research may be oriented towards investigating what makes a situation pathogenic, rather than whether a bacterium is pathogenic.

## Data Availability

No data were generated in this work.

## Note

1. The experiments suggested here were inspired by personal communication with Bernhard Krismer, Institute for Microbiology and Infection Medicine, University of Tübingen, Germany.
